# Flowrate Sensing and Measurement in Portable Smart Inhalers

**DOI:** 10.3390/s24216848

**Published:** 2024-10-25

**Authors:** Ivan Mysovskikh, Mathew Legg, Serge Demidenko

**Affiliations:** 1 College of Sciences, Massey University, Auckland 0632, New Zealand; imysovskikh@outlook.com (I.M.); mathew@nzse.ac.nz (M.L.); 2 New Zealand Skills and Education College, Auckland 1010, New Zealand; 3 School of Engineering & Technology, Sunway University, Bandar Sunway 47500, Malaysia

**Keywords:** review, smart inhaler, inhalation flowrate, airflow measurement

## Abstract

This review discusses approaches and implementations of flowrate sensing and measurement in smart inhalers for effective respiratory disease management. It highlights the importance of compliance with proper inhaling techniques and consistent adherence for managing respiratory conditions. Methods and relevant commercial and prototype research-type devices for sensing and measuring inhalation flowrate in smart inhalers are studied and compared. The study argues that the utilisation of acoustic analysis and air-pressure sensing is a promising approach to detect and evaluate the inhaling action, ultimately allowing improvement in the treatment outcomes and life quality of patients with respiratory diseases.

## 1. Introduction

Chronic Obstructive Respiratory Diseases (COPDs), including asthma, affect millions of people worldwide. Asthma symptoms include breathing difficulty, wheezing, coughing, and chest tightness. In severe cases, it can result in hospitalisation or even death. Asthma also impairs the quality of life of patients, their families, and their caregivers. For instance, New Zealand faces a particularly serious asthma challenge, with approximately one-eighth of its population (or over 597,000 people) suffering from the disease [1,2]. The country has a high incidence of asthma among children, who account for the majority of hospitalisations due to asthma [3]. Furthermore, asthma disproportionately affects native Māori and Pacifica people, who experience higher rates of hospitalisation and mortality from asthma and other respiratory diseases than other ethnic groups. Furthermore, asthma imposes a significant negative economic impact on the national economy, costing approximately NZD 7 billion per year in direct and indirect expenses [3].

Asthma is a chronic condition that demands continuous management and education for patients. In many cases, people with asthma lack adequate knowledge to control the disease symptoms. Often, they also fail to use portable medication application devices (so-called inhalers) properly or consistently [1]. The main purpose of the inhaler is to deliver aerosol medication to the lungs. However, the use of a correct technique is required to make the application effective. Incorrect use of an inhaler device (misuse) includes a variety of errors, such as not shaking the device before use, not inhaling deeply enough, or not holding the breath long enough after inhaling the medication. In addition, it is important how consistently a patient follows the prescribed inhalation regime (adherence). Both misuse and adherence are critical for effective respiratory disease management.

Indeed, improper application of the inhaler could lead to aggravating asthma conditions. This ultimately negatively affects the treatment efficiency and leads to higher cost [4,5,6]. Notably, the misuse of inhaler devices in clinical practice was reported to be independent of patients’ clinical or functional characteristics.

Inhaler misuse is often caused by deficiencies in relevant knowledge, incorrect interpretation of printed instructions, or users’ training by healthcare professionals. The correctness of the employed inhaler technique should be regularly verified and reinforced. However, this is frequently impractical in clinical settings. This issue can be addressed by incorporating relevant sensing, evaluation, monitoring, and feedback facilities within inhalers themselves or as add-ons, thus making them smart devices. Among other functions, smart inhalers can gather usage data and indicate whether or not the inhaler is being used correctly. Such devices are capable of logging the intensity of usage, checking proper medication load, detecting inhalation and exhalation timings, notifying if the device is not shaken before use, etc. However, most smart inhalers in the market do not evaluate the quality of the inhalation process in detail [7,8]. The inhalation quality largely depends on the inhalation flowrate, which is the volume of air–medication mixture that flows through the inhaler per unit of time (usually measured in litres per minute or L/m). Therefore, a consistent inhalation flowrate for a certain duration of time is crucial for the optimal depositing of the medication in the lungs.

Several smart inhalers and add-on devices are available on the market, offering various functionalities, such as tracking and logging medication usage, providing reminders for medication intake, etc. There are also literature reviews that cover various aspects of smart inhaler research, technology, and implementation, e.g., [9,10,11,12]. This paper provides an additional contribution to the body of knowledge in the field. To the best of the authors’ knowledge, it offers the first review study focusing specifically on smart inhalers having flowrate sensing and measurement capability. It underlines the significance of inhalation flowrate measurement and control, investigates the current progress in the field, and identifies promising directions for future research and development.

The article is structured as follows. Section 2 describes inhalation flowrate, demonstrates its role in medication deposition, and highlights its significance in COPD therapy. Section 3 presents solutions for training users on the improved inhaler technique for proper medication inhalation. Then, Section 4 describes a range of devices designed to enable real-time inhalation flowrate measurement. Finally, the conclusion is given in Section 5, which summarises the devices and solutions discussed in the review.

## 2. Inhalation Flowrate

The right flowrate is crucial for patients with asthma and COPDs as it determines the delivery and deposition of inhaled medications [13,14,15]. Insufficient inhalation flowrate leads to lower drug emissions. It is particularly relevant for Dry Powder Inhalers (DPIs). In reference [16], the authors emphasized that different inhalers, such as Pressurised Metered-Dose Inhalers (pMDIs) and DPIs, need different inhalation flowrates to deliver the drug effectively. Different inhalers were used to examine the Peak Inspiratory Flowrate (PIFR) at different levels of device resistance. It was found that a significant number of patients could not reach the minimum or optimal PIFR for DPIs. A similar result was observed in [17]. Another study [18] compared the performance of six different DPIs (three types each of two strength levels) and found that the fine particle dose flowrate dependence was similar for all inhalers. A new metric to measure the flowrate dependence on lung dose was proposed in a detailed review of DPIs [19]. Among others, the study showed that lung dose depended more on the formulation and device characteristics than on the flowrate. The authors claimed that with changes in formulation and/or device characteristics, passive DPIs could deliver the drug regardless of inhalation technique. One literature review [6] highlighted the differences in optimal inhalation techniques among inhalation devices. Different inhalers have varying resistance requirements for inhalation flow. For instance, pMDIs and SMIs do not require a strong flow from patients. Instead, they are designed for slow, deep, and long inhalations. On the other hand, the inhalation resistance of DPI inhalers may vary from one device to another, introducing low, medium, and high-resistance devices. This provides a choice for patients with limited lung function to use the device with an appropriate optimal flowrate. It was noted that healthcare providers should be sure their patients are capable of achieving the required inhalation flowrate, which could vary for device types and models. For instance, it was shown in [20] that, in some cases, low-resistance DPIs could achieve higher and more consistent lung deposition than high-resistance DPIs. It suggested that low-resistance DPIs might be more suitable for COPD patients of different severities.

On the other hand, reducing inhalation airflow may also have a positive effect in some cases. As it was noted in [21], too-high aerosol speed from a pMDI or too-high inhalation flows from some DPIs may cause excessive droplet deposition in the oropharynx and lower lung regions. In [22], the authors compared the inhalation dosimetry in the respiratory tract for five breathing depths. They observed significant differences in airflow dynamics and deposition distribution in the oral cavity. The inhalation depth was positively correlated with mouth deposition and negatively correlated with small airway deposition. The optimal delivery efficiency to small airways was achieved at quite low flowrate and decreased with higher inhalation depths.

The size, speed, and behaviour of the aerosol droplets or particles that carry the drug are also affected by the inhalation flowrate. This influences how deep particles can penetrate into the lungs and where they can be deposited. In [23], a computational fluid dynamics model was developed to characterise aerosol flow from a pMDI into a simulated mouth–throat geometry and to analyse the effects of air flowrate and cone angle. The model showed that increasing flowrate had various effects, such as particle deagglomeration and throat particle deposition reduction. The model also indicated that particle deposition depended more on the initial cone angle than on the flowrate and that an 8-degree cone angle was optimal for the lowest mouth–throat deposition. Another study [24] confirmed in vivo that the total and regional lung depositions were determined by the particle size of the aerosol and the inhalation flowrate, rather than the device resistance.

Following the deposition topic, in [25] the authors used a model based on a realistic geometry of the human airway derived from computed tomography to study the effects of various factors on the deposition. They discovered that higher inhalation flowrate increased the turbulence and the particle deposition in the main bronchi, especially for larger particles. It was also observed that impaction was the dominant deposition mechanism for larger particles at higher flowrates, rather than electrostatic deposition.

Various aspects (and relevant errors) of inhaler application must be considered. Among them is the timing/volume of drug release. This is critical for optimal lung deposition, as the drug must be dispensed within the best window of the inhalation cycle [26]. The article highlighted the importance of determining the inhalation flowrate profile, as it enables the estimation of key inhalation parameters such as inhalation depth and duration, exhalation before inhalation, and lung function.

Study [27] clarified that the main parameter for DPI performance was not PIFR but the negative pressure generated by the patient’s inhalation effort. The authors investigated the factors influencing inspiratory pressures and flowrates, along with the dispensing and dispersion characteristics of different DPIs. They discovered that inspiratory pressures (not flowrates) constrained and regulated the patient’s ability to generate enough flow for effective DPI use. The article also emphasised that employed inhalers had different operating mechanisms and resistances.

It is important to reiterate that the inhalation flowrate is a significant factor determining the efficacy and safety of inhalation therapy. However, many patients with asthma and COPDs cannot achieve a suitable inhalation flowrate for their inhalers, especially during acute exacerbations. This may result in suboptimal therapy and poor clinical outcomes [16]. As shown in [28,29], inhaler mishandling is still a major clinical issue. Moreover, it was reported in [17] that changing the inhaler type was sometimes associated with an increase in the number of errors. Inhalation techniques should be taught and practised to help patients to optimise their inhalation therapy. However, compliance with the correct technique declines over time [30], highlighting the high value of regular assessment and monitoring. It is important to mention that clinical practice is not the only way to train patients. For instance, an in vivo study [31] showed the effectiveness of using inhaler add-on devices with smartphone applications for adults. Such devices enhance the pMDI inhalation technique through audio–visual feedback, thus leading to improved salbutamol delivery to the lungs. Furthermore, it was shown that slowing the inhalation step was strongly associated with the amount of medicine that reached the lungs and excreted in the urine.

## 3. Inhalation Technique Training

Various simulators are available on the market and employed in medical practice to train patients to correctly regulate inspiratory flowrate. Such training devices do not require relevant medical authority approvals as they are not intended to be used for medication delivery purposes. Several review studies of different training devices and their efficiency have been conducted, e.g., [21,32]. This section focuses on the devices that aim to real-time control inhalation flowrate. The goal is to identify potential areas for improvement. The section excludes the bulky spirometers or Aerosol Inhalation Monitors (AIM) used in hospitals, as they are not pertinent to the current topic of portable inhaler devices for routine use.

### 3.1. 2Tone Inhaler Trainer

The 2Tone Inhaler Trainer is a commercially available device developed by Canday Medical Ltd. (Newmarket, Suffolk, UK) that teaches patients how to use their MDIs correctly. The device resembles a real MDI in shape and feel. It does not contain a medication canister as it is employed for training only. The design of the device is simple. It emits sounds (tones) depending on the speed of inhaling. Silence indicates too slow breathing. The presence of two different tones indicates too-fast breathing, while the advent of just one tone indicates optimal breathing. According to the online brochure, practising with the sound feedback device enables patients to quickly learn to adjust their inhalation speed and improve their inhaler technique [33]. As a result, patients should get used to breathing through their real inhaler deeply at the right speed.

Only a few studies are available on the effectiveness of the 2Tone Inhaler Trainer, such as references [34,35,36]. The studies concur that the 2Tone trainer can improve the inhalation technique by helping to maintain a slow inhalation rate when using an MDI. They also report increased asthma patients’ satisfaction and improved confidence with their inhaler use. One study [37] compared the efficacy of using the 2Tone device with only verbal training for achieving a slow inhalation flow. The study shows that the 2Tone device has an advantage over verbal training and positively impacts the patient inhalation technique. However, it is noteworthy that the device is based on using a sound signal, which could be inconvenient for regular use and unsuitable for deaf or hearing-impaired patients.

### 3.2. Clement Clarke pMDI Add-Ons

The medical supplier company Clement Clarke (Bury St Edmunds, Suffolk, UK) offers several commercially available inhaler add-on devices that help patients improve their inhaler technique for pMDI by optimizing their inhalation flowrate [38]. All the devices have a similar functionality.

The first device is Flo-Tone, a trainer that helps patients to achieve the correct flowrate and coordinate actuation. The device can be attached to the mouthpiece of a placebo pMDI or Trainhaler, which is a mock pMDI enclosure with a canister pressing imitation. Flo-Tone has an integrated whistle that produces a sound when the patient reaches the target inspiratory flowrate. After actuation, the whistle helps to maintain a slow inhalation to deliver the medication to the lungs. For a better experience, the device can be used with the “Trainhaler Buddy” mobile application. It recognises the whistling sound and provides real-time feedback with graphical visualisation. This helps patients to see if they have achieved the optimal inhalation technique and the duration of inhalation in seconds. It is important to note that the device has low accuracy. The device can be attached to a regular pMDI. However, using it only with a placebo pMDI or Trainhaler is recommended. This might be because the effect of the whistle by-pass channel might influence inhalation outcomes such as aerosol droplet size and deposition.

The second device is the Flo-Tone CR, which is an upgraded version of the previous device. It has a built-in Controlled Resistance (CR) that eliminates the inhaler’s internal resistance variability. This enables the device to fit all pMDIs since the whistle indicates the same flowrate regardless of the pMDIs’ resistance. It also has a tethered mouthpiece cap. It is claimed that using the device as a spacer enhances drug delivery and reduces unwanted oropharyngeal deposition.

Another add-on device aiming to improve inhaler technique is the Clip Tone E. It has a different design but the same functionality and purpose as the above-described devices. It produces a whistle when the user inhales slowly and steadily. Instead of the mouthpiece, it fits around the pMDI canister at the top of the inhaler enclosure. This design prevents the add-on from affecting medication delivery, as it is away from the medicine injection path. However, the device has limited use, as it is compatible only with a few specific models of pMDI.

The efficacy of Trainhaler and Flo-Tone CR training devices is assessed in [39]. The study involved 64 asthmatic patients on pMDI therapy for at least a month. They exhibited a poor pMDI technique, including a too-fast inhalation flowrate. The results indicate that Trainhaler and Flo-Tone CR enabled enhanced inhalation with effective flowrate control. The devices also allow the patients to practice at their own pace and time.

A broader study [40] employed Trainhaler combined with Flo-Tone to examine the effectiveness of the inhalation technique training. It involved 304 adult asthma patients divided into two groups (intervention and base). The patients of the intervention group utilized the inhalation technique three times, with a one-month gap between each performance. At each visit, their pulmonary function and inhalation technique were assessed. This was then followed by feedback and guidance on the correct technique application. The intervention group showed a significant reduction in the frequency of the crucial errors one visit earlier compared to the base group subjected to only verbal counselling.

The effect of advanced patient consulting supported by two similar devices and a smartphone application is examined in [41]. The study engaged 100 children—asthma patients. The pMDI was used together with a whistling device and inhalation spacer—a transparent chamber fitted between the inhaler and the mouthpiece. The spacer allowed monitoring of the intake and could be used to see if all the medication had been inhaled. The whistling devices included the Flo-Tone and a facemask with a built-in whistle tone that could be detected by the Trainhaler smartphone application. The patients were assigned into two groups based on the type of training device they used. The study evaluated the inhalation technique three times, with one-month intervals. After each evaluation, the patients received a training session. The results showed a significant reduction in the number of mistakes for both groups. However, the Flo-Tone-associated group achieved the desired increase in inhalation duration earlier. The facemask-associated group experienced difficulties in developing the proper technique, especially among the younger patients.

Study [42] provides a comparison of traditional verbal consulting and the advanced method employing the Flo-Tone with the mobile application. In total, 201 asthmatic children were randomly assigned to two groups (control and advanced). They demonstrated their inhalation technique three times at one-month intervals and received a training session after each demonstration. The advanced group exhibited a faster and greater decrease in the number of mistakes and an increase in inhalation duration than the control group. Moreover, the advanced group also displayed a higher and quicker improvement in lung function than the control group. The authors noted that the combination of traditional (verbal) and advanced consulting methods led to significant enhancements in inhalation technique and duration compared to using only traditional verbal consulting. The study has been replicated in [43] with a smaller number of subjects and yielded similar findings.

The effectiveness of advanced consulting of adult subjects is discussed in [44]. Flo-Tone CR and Clip-Tone E were used with a smartphone application. The outcome was compared with the results of the regular inhaler training. Twelve healthy adult volunteers participated in a cross-over study. They were trained to use the following: (1) pMDI alone, (2) pMDI with the Clip-Tone E add-on, and (3) pMDI with the Flo-Tone CR add-on. They received guiding materials and personal training for each device. The participants used each device twice during a session. The amounts of medicine delivered during inhalation were estimated by urine samples taken 30 min past inhalation. The results revealed that the use of the add-on devices led to a significant increase in the concentration of the medicine in the urine samples compared to using the pMDI alone. At the same time, no significant differences were registered 24 h after the inhalation. Overall, the study concluded that the advanced methods significantly enhanced the inhalation technique for adults.

The above-presented findings are confirmed in [31]. The study followed the same pattern but with a larger number of subjects (316 asthma patients). The patients were split into three groups. Two of them used the add-on devices while the last one used the pMDI alone. Each group had three sessions of technique demonstration and training. The results showed that the add-on devices, with their visual and auditory feedback, allowed better engagement of the patients while improving their inhalation technique and lung function. The Flo-Tone CR and Clip-Tone E user groups outperformed the control group significantly in terms of the lower number of mistakes in the technique. However, the authors did not find any significant difference between the efficiency of the two add-on devices.

### 3.3. DPI Whistling Trainers

Some of the DPIs have mock copies available, such as the Turbuhaler (AstraZeneca AB, Södertälje, Sweden), Accuhaler (GlaxoSmithKline, Brentford, Middlesex, UK)—the same as for Diskus inhaler, Ellipta (GlaxoSmithKline, Brentford, Middlesex, UK), and others. These trainers have a whistling capability. The whistling is triggered when reaching the target inhalation flowrate. However, the whistle sound is usually quite loud. Thus, it is intended only for training purposes. Unlike the research activity for pMDI, no research on the effectiveness of training devices for DPI has been reported in recent years. The results of pMDI-focused studies cannot be generalized to DPI, as they have completely different inhalation flowrates and technique characteristics. This can be attributed to the different flowrate values since DPIs require fast and deep inhalation. A different metric, such as pressure drop [27], should be used instead of maintaining slow and long inhalation typical for pMDI.

The devices reviewed in this section can improve the inhaler technique and overall treatment outcomes for patients who have respiratory diseases. Unfortunately, they have drawbacks requiring attention, such as inconvenience in private use and compatibility with different medications. The choice and use of inhalers still depend on consultation with healthcare providers. This indicates that further research is necessary to design and test new generations of smart devices that offer more precise inhalation flowrate measurement and provide personalized feedback to the users.

## 4. Inhalation Flowrate Measurement

Various products have been developed and implemented to measure and control the inhalation flowrate. This section outlines the most notable of these devices that utilise acoustic and pressure sensors to enhance inhaler technique and adherence. The state of development, benefits, drawbacks, and commercialisation potential of these devices are discussed.

### 4.1. Inhaler Compliance Assessment Device

A group of Irish researchers from Trinity College (Dublin, Ireland) conducted a series of studies on the acoustic analysis of DPI use. They developed a device and a method to record and evaluate the sounds produced by DPIs to assess patients’ adherence to the correct inhalation technique. The initial investigation, published in [45], involved 12 asthma patients who attended a respiratory clinic for three months. An acoustic pickup device combined with acoustic signal analysis was used to achieve inhalation detection with 89% accuracy. However, the solution faced challenges such as artefact interference and offset time determination due to the flowrate variations at the end of inhalation.

Based on these promising results, the study was extended by taking into account the correlations between flow/volume parameters and the acoustic measurements. The device was named Inhaler Compliance Assessment (INCA). The main objective of the extended study was to explore the possibility of using acoustic signals to measure the inspiratory flowrate and drug delivery from the DPI. An in vitro study [46] was performed using a vacuum pump to simulate different inspiratory efforts. Two more studies [47,48] were carried out using volunteers to collect acoustic and flowrate records. It was found that several acoustic parameters had strong correlations with PIFR. Also, an algorithm was proposed for identifying the inhalation and exhalation events with up to 92.8% accuracy. Unlike the earlier research focused only on respiratory sound signals, the reported studies analysed both the respiratory and inhaler device sounds.

Paper [49] reports the extension of the study where the PIFR and the Inspiratory Capacity (IC) were quantitively estimated using the acoustic features of inhalations. Acoustic signals were captured and analysed by the INCA device. The PIFR was found to correlate with the average power in certain frequency bands and with the amplitude parameters of the inhalation signal, such as median, mean absolute deviation, and root mean square. The results showed that the average power in the 300–600 Hz frequency band is strongly correlated with PIFR and IC for the *INCA* device with Diskus. Therefore, the study proved the possibility of employing acoustics to monitor inhaling objectively.

The research was further extended and reported in [50,51]. The main goal was to discover an objective method to evaluate the inhalation technique without relying on subjective judgments. An algorithm was developed and deployed to automatically detect inhalation from audio signals while providing feedback on patient adherence to the expected technique. It achieved an 83% accuracy rate in evaluating the inhaler technique. The algorithm could identify major critical errors such as exhaling into the mouthpiece, insufficient inhalation force, and multiple inhalations. Also, it enabled the continuous monitoring of patient competency in a home environment, not just in the clinical setting. The extension study is reported in [52]. A larger group of patients was engaged to examine how the inhalation flowrate and duration affect the aerosol delivery.

The proposed method can be applied to other DPIs (e.g., Turbuhaler and Evohaler) [53]. The mean acoustic power was found to be the most reliable predictor of PIFR for all of the inhalers tested. However, some studies note the limitation of recording acoustic signals in a noise-free environment (e.g., [50,53]) as not representing realistic acoustic conditions. To address the limitation, it was suggested to concentrate on the orientation and number of microphones, along with the use of adaptive noise cancellation.

The INCA Sun Research Study [54,55] was an in vivo clinical trial that examined the INCA device’s effect on managing severe asthma. It involved 200 adult patients suffering from poor asthma control [56]. The trial was conducted from October 2015 to January 2020. The study aimed to enhance inhaler therapy adherence and asthma control by comparing treatment decisions based on digitally acquired data with those based on conventional methods. Patients were randomly assigned to either the active or control group. Both groups attended three education visits over 8 weeks. This was followed by three treatment adjustment visits over 24 weeks. Digital inhaler adherence data guided treatment adjustment of the active group, while the control group used traditional methods. A third of the active group had dose reductions without increased airway inflammation, symptoms, or exacerbations, thus lowering side effect risks. This reduction did not compromise asthma control. The digital clinical decision support tool helped clinicians adhere to treatment recommendations. Medication adherence was improved by approximately 10%, with a significant reduction in the treatment volume. This illustrates that digitally aided asthma care can be cost-effective and clinically beneficial.

Another similar two-stage study [57] showed that the INCA device improves adherence and inhaler technique when used during consultations. Furthermore, the patients were satisfied with the device and the consultations. Despite the smaller sample size (18 adult COPD and asthma patients), the study demonstrates that embedding an objective measure of adherence and inhaler technique during community pharmacists’ consultations enhances the patients’ adherence and inhaler technique.

### 4.2. Sagentia Innovation VeriHaler

The VeriHaler is an acoustic device by Sagentia Innovation (Harston, Cambridge, UK) that aims to improve patient adherence to inhaler therapy [54]. It features a built-in microphone that records the acoustic signals of inhalation and delivers feedback via a mobile application. The device is compatible with pMDIs and DPIs. It has a non-intrusive electronic design that does not affect the medication delivery. VeriHaler eliminates background noise and accurately measures flowrate and inhaler usage. It promises to detect key inhalation parameters such as PIFR, inhalation timing, and formulation delivery. The mobile application enhances the user experience by enabling communication with the healthcare provider and alerting the patient in case of any signs of deterioration in their condition or employed technique. The device is still in the development stage.

### 4.3. Tone Elements

An acoustic dry powder inhaler that could measure inhalation flow and medication dose was developed by the University of Copenhagen (Copenhagen, Denmark) and presented in [58]. The device employs a passive acoustic component with comb-shaped teeth to produce sound signals changing with the air flowrate. The flow parameters associated with the inhalation technique are obtained by examining the features of the sound signals (e.g., amplitude, frequency, etc.). It is noteworthy that the device has a two-inlet design. Only one of them employs a passive acoustic component. This lowers the breathing resistance. This is particularly important for patients with severe airflow obstruction. The study investigated the effects of different geometries of acoustic elements. The results show that the proposed design with a single-tone gap does not produce clear and distinct spectral peaks indicating tone components. On the other hand, increasing the number of gaps improves the resolution of the spectra and the correlation with the airflow rate. At the same time, increasing the distance between the gaps reduces the distinctness of the spectral peaks at lower frequencies. Moreover, the combination of two larger gaps and increasing gap distance results in less pronounced and distinct spectral peaks.

The design of the tone element was not justified or compared with any existing topologies. Also, the analysis was limited to the human audible range (20–20,000 Hz), without explaining why this was done or if the device generated tonal signals outside this region. A device that generates an ultrasonic tone (above 20 kHz) might be beneficial as it would be outside the frequency range of most background noise. Furthermore, it would be preferable to have an inaudible acoustic element for the final product, as the device should not disturb other people with its noise. Despite the potential of the device for medication tracking for asthma and COPD patients (aiming to enhance their disease management and improve inhaler techniques), the project was not followed up or commercialized.

### 4.4. Amiko Respiro

Respiro is a digital platform created by Amiko (London, UK). It offers electronic solutions to track and record inhaler usage. The platform is compatible with various inhalers. The only currently commercially available model is RS01X. It incorporates built-in sensors and digital features [55]. The platform uses machine learning and sensing to measure and analyse the inhalation techniques, including inspiratory effort and inhalation flowrate. These metrics are assessed using pressure-drop measurements, enabling the real-time estimation of the inhalation technique [59,60]. The data can be synchronized with a mobile application, which acts as a companion to the inhaler. It reminds a patient when to take a dose. It also provides personalized feedback and suggestions based on acquired data while using artificial intelligence to help a patient self-manage the use of the tool more effectively. Furthermore, it allows data sharing with healthcare providers. Unfortunately, no information was found in the literature presenting the in vivo tests of the RS01X device and its comparison with conventional methods of sustaining inhaler technique adherence. Nonetheless, the successful use of the platform with the Ellipta inhaler was reported in [61]. The patients showed high adherence to inhaled medication. They also completed daily diaries as part of the self-management intervention.

### 4.5. Intelligent Control Inhaler

The Intelligent Control Inhaler (ICI) is a smart device developed by the 3M Drug Delivery Systems (St. Paul, MN, USA) to enhance the treatment of respiratory diseases. The device was introduced in 2016 [62]. It received the top award in a new category at the CPhI Pharma Awards that year [63]. The device evaluates the inhalation technique and monitors drug delivery. It can also regulate the inhalation flowrate. It is equipped with an integrated screen providing user instructions and feedback. The device offers a mobile application allowing users to share data with their healthcare providers via an inhalation data management platform. It employs a combination of breath actuation and innovative technology to control inhalation flowrate and automatically improve the consistency of drug delivery. It is assumed that the device achieves this control function by varying its inhalation resistance characteristics [64]. The latest update [65] states that the device is still under development by Kindeva (St. Paul, MN, USA), formerly 3M Drug Delivery Systems.

### 4.6. Teva Digihaler

Another option for inhalation profile evaluation is the use of the Digihaler family of smart inhalers developed by Teva Respiratory (Parsippany, NJ, USA) [66]. These devices have been approved by the Food and Drug Administration (FDA). Three models of Digihaler devices having the same shape and electronic platform but differing in their medication were developed. The first model is the ProAir Digihaler. It is a rescue inhaler that contains a short-acting medication to relieve or prevent bronchospasms associated with asthma and COPDs as well as exercise-induced bronchospasms. The ArmonAir Digihaler is a maintenance inhaler for long-term treatment that reduces inflammation and swelling of the airways. The AirDuo Digihaler is also a maintenance device that contains a long-acting medication for controlling symptoms such as wheezing and airway narrowing. The Digihaler devices are equipped with a pressure sensor, wireless transmitter, and processor. The pressure sensor is connected to a mouthpiece. It senses all inhaler activities. The device estimates the inhalation flowrate in real time using pressure measurement. The device can be paired with a smartphone app to track inhaler usage data, such as peak inspiratory flowrate, inhalation volume, and events. The data can be shared with healthcare providers. Additionally, it offers feedback to help users improve their inhalation technique, potentially preventing symptom worsening.

A predictive model for Clinical Asthma Exacerbation (CAE) based on data collected from the ProAir Digihaler was developed and validated in a pilot study involving 360 poorly controlled asthma patients [67]. The model used metrics such as PIFR, volume, duration, and time-to-peak flow as predictors. The results showed that the average number of daily inhalations over five days is a reliable indicator of CAE risk. The same data were later used in another study [68] to build a machine-learning model for CAE prediction. The machine-learning model also confirmed that increased medication consumption is a predictor of CAE events and can forecast their occurrence within the next five days.

The efficacy of the ProAir Digihaler was demonstrated in a randomized controlled trial [69]. The main objective of the trial was to compare the ProAir Digihaler application with standard asthma care. A total of 333 participants were randomly assigned to either the Digihaler enhanced or the standard care group. Their inhalation data were monitored weekly for 12 weeks. The trial found that participants using the Digihaler device had an 85.3% higher probability of improving their asthma control than those using standard care.

However, there is also a downside to the Digihaler devices. All the devices have a dose counter attached to the actuator. It shows the number of remaining doses. Since these devices are all-in-one, they are to be discarded once they run out of doses. Moreover, they are relatively expensive.

### 4.7. Sensirion Inhaler Clip-On

A prototype of a clip-on device with a pressure sensor has been designed and patented by Sensirion (Stäfa, Switzerland) [26,70]. It is attached to the inhaler and measures the patient’s inhalation airflow and timing of the inhaler actuation. The device employs a differential pressure sensor enabling the measurement of mass flow in a bypass configuration. The bypass configuration preserves the flow path of the inhaler and ensures no interference with the existing inhaler device function. This feature offers a significant benefit as it eliminates the need for revalidation of the inhaler with the FDA.

The inventors refer to the Bernoulli/Venturi effect to describe how the patient’s inhalation causes the air flow entering the inhaler to accelerate and create a negative pressure at the inhaler top opening (around the canister) and the opening of the clip-on device. The negative pressure magnitude is directly proportional to the flowrate magnitude of the airflow through the inhaler to the patient and is detected by the flow sensor. By measuring the bypass airflow and knowing the bypass-to-main-pass ratio (which is determined by the clip-on/inhaler geometries), the total airflow to the patient can be calculated. The device uses the world’s smallest differential pressure sensor from the Sensirion SDP3x family. This solution is optimal for measuring mass flow in a bypass configuration. Unfortunately, it is relatively costly, thus affecting the device’s accessibility. However, it has the advantage of being reusable (unlike the Digihaler devices discussed earlier). Currently, the device has no data on experimental testing or research on its efficacy. It is also not yet available on the market while having a trademark.

### 4.8. Adherium Hailie

Hailie sensors have been developed by Adherium Limited (Auckland, New Zealand). They can be attached to different inhalers. The sensors are linked to a phone and record medication actuation events. Initially, they did not measure the inspiratory flow. However, the next generation of devices is capable of measuring flowrate and detecting inspiratory flow, inhaler shakes, and inhaler orientation. Inhalers such as Trava ProAir, Advair, Ventolin, and Symbicort are supported by the Hailie devices. The FDA approval for these next-generation sensors was obtained in late 2021 [71] and received FDA clearance later [71]. Unfortunately, no results of clinical studies of the devices and their benefits were found in the literature.

### 4.9. CapMedic

CapMedic is a digital add-on device designed by Cognita Labs (Santa Cruz, CA, USA) to assist patients with asthma and COPDs to use pMDIs correctly. It provides real-time feedback by audio–visual cues throughout the steps of the device application. Spirometry and measurement of lung exhalation parameters such as Forced Expiratory Volume and Peak Expiratory Flow are among the declared features [72]. These measurements are performed using built-in sensors. They provide users with information on their actual inhalation technique.

CapMedic can be beneficial in improving the inhaler application technique and medication delivery as indicated by the publications [73,74]. However, significantly more substantial independent studies are needed to fully validate the efficiency of the device. Furthermore, the technical details of the sensors are not well-documented in the literature. In addition, the CapMedic device operates while employing a subscription-based model, which may pose a barrier for some users.

## 5. Conclusions

The significance of the inhalation flowrate measurements and the current state of the related technologies are reviewed in this paper. The review demonstrates that proper inhaler technique and consistent adherence are crucial for effectively managing respiratory conditions. Monitoring inhalation flowrates emerges as a promising approach to assess the progress. However, the widespread mishandling of inhaler application techniques poses a significant clinical challenge. This is particularly true in ensuring the accurate dose delivery, which depends on the optimal flowrate. Unfortunately, this is specific to an inhaler model.

Results in the relevant recent literature support special training device engagement to guide and improve inhalation techniques. However, despite the demonstrated positive impact, training inhalation devices have limitations such as inconvenience in private usage and incompatibility with different medications and inhaler models. The ongoing reliance on healthcare professionals for inhaler selection and utilization highlights the need for research to develop smart inhaler devices capable of flowrate measurements and providing personalized feedback. The development and application of smart inhaler technologies and products (particularly those facilitating flowrate control) have become a critically important task.

The integration of smart inhalers, add-ons, and training devices has demonstrated promising positive impacts on COPD treatment outcomes in recent years. Indeed, the technologies have not reached their final form, and it is likely that in the near future such devices will receive wider usage and application among patients. This could be driven with a practical design approach, such as having sensors and electronics that could be used multiple times, universal device shape suiting different medication containers, free mobile companion application, etc. On the other hand, such technology advancements should not be limited to only one manufacturer and medication type but rather be widely available for patients in terms of price and availability. Based on current trends with the uptake of smart devices, tiny sensors, and cheap electronics, access to smart inhalers should not be a privilege but a standard healthcare practice.

The presented review on existing and forthcoming new perspective solutions in the field of inhalation flowrate measurement aims to contribute towards addressing this task. Table 1 and Table 2 summarise information on the smart inhaler devices that are used, respectively, for training and flowrate measurement that are referred to in this review. Most of the flowrate measurement devices are not yet commercially available.

Approaches based on the acoustic analysis of the inhalation process have been introduced, using cost-effective acoustic sensors. Despite the strong potential, none of such devices have reached the commercial availability stage, with only one passing through some trials after extensive development. This highlights the need for further research and trials to overcome the challenges and limitations of such a novel approach.

## Figures and Tables

**Table 1:** Comparison of the inhalation training devices.

Device / Approach	Developer	Status	Device Type	Key Features	Advantages and Drawbacks
2Tone Inhaler Trainer	Canday Medical Ltd., Newmarket, Suffolk, UK	Commercially available	pMDI Trainer	Passive acoustic component	(+) Simple construction, provides real-time and clear feedback on the PIFR (–) Does not deliver medicine, does not take measurements, noisy
Flo-Tone, Flo-Tone CR, Clip-on	Clement Clarke, Bury St Edmunds, Suffolk, UK	Commercially available	pMDI Trainer	Passive acoustic component	(+) Simple construction, provides real-time and clear feedback on the PIFR (–) Does not deliver medicine, does not take measurements, noisy
Turbuhaler Training Device	AstraZeneca AB, Södertälje, Sweden	Commercially available	DPI Trainer	Passive acoustic component	(+) Simple construction, provides real-time and clear feedback on the PIFR (–) Does not deliver medicine, does not take measurements, noisy
Accuhaler Inhalation Training Device	Glaxo Group Ltd., Brentford, Middlesex, UK	Commercially available	DPI Trainer	Passive acoustic component	(+) Simple construction, provides real-time and clear feedback on the PIFR (–) Does not deliver medicine, does not take measurements, noisy
Ellipta Inhalation Trainer	Glaxo Group Ltd., Brentford, Middlesex, UK	Commercially available	DPI Trainer	Passive acoustic component	(+) Simple construction, provides real-time and clear feedback on the PIFR (–) Does not deliver medicine, does not take measurements, noisy

**Table 2:** Comparison of the existing solutions in inhalation flowrate measurement.

Device / Approach	Developer	Status	Device Type	Key Features	Advantages and Drawbacks
INCA	INCA Team	Passed in-vivo trials, but not available commercially	DPI	Acoustic analysis	(+) High accuracy in event detection (–) Challenges with artifact interference
VeriHaler	Sagentia Innovation, Harston, Cambridge, UK	Under development and trials	pMDI and DPI	Acoustic analysis, Noise cancellation algorithm	(+) Platform works with both pMDI and DPI (–) Still in development, no design details and in-vivo test data
Tone Elements	University of Copenhagen, Copenhagen, Denmark	Prototyped concept	DPI	Passive acoustic component	(+) Good correlation with airflow rate (–) Requires changes in the airflow passage and FDA approval
Amiko Respiro RS01X	Berry Global Inc., Evansville, IN, USA	Commercially available	DPI	Pressure sensor, Machine Learning	(+) Platform works with a variety of inhalers (–) No in-vivo test data available, no other device within platform commercially available
Intelligent Control Inhaler	Kindeva Drug Delivery, St. Paul, MN, USA	Under development	Unknown, but sketches refer to DPI	Regulates inhalation flowrate	(+) Integrated screen, mobile app (–) Not yet launched, no research activity
Digihaler	Teva Respiratory, Parsippany, NJ, USA	Commercially available but discontinue soon	DPI	Pressure sensor, FDA approved	(+) The only robust and efficiency-proved solution available commercially (–) Expensive, single-use design
Inhaler Clip-on	Sensirion AG, Stäfa, Switzerland	Patented, but not available commercially	pMDI	Pressure sensor, does not require FDA approval	(+) No interference with inhaler function (–) Costly sensor, no in-vivo test data available
Adherium’s Hailie	Adherium Ltd., Auckland, New Zealand	Received FDA clearance	pMDI	Unknown	(+) Based on robust existing platform for add-ons without physiological measures (–) Not yet launched
CapMedic	Cognita Labs, Santa Cruz, CA, USA	Commercially available	pMDI	Spirometer	(+) Clinical grade accuracy declared (–) Subscription-only service, not many evidence of device efficiency
